# Brain stimulation modulates driving behavior

**DOI:** 10.1186/1744-9081-4-34

**Published:** 2008-08-06

**Authors:** Gian Beeli, Susan Koeneke, Katja Gasser, Lutz Jancke

**Affiliations:** 1 University of Zurich, Institute of Psychology, Division Neuropsychology, Switzerland

## Abstract

**Background:**

Driving a car is a complex task requiring coordinated functioning of distributed brain regions. Controlled and safe driving depends on the integrity of the dorsolateral prefrontal cortex (DLPFC), a brain region, which has been shown to mature in late adolescence.

**Methods:**

In this study, driving performance of twenty-four male participants was tested in a high-end driving simulator before and after the application of transcranial direct current stimulation (tDCS) for 15 minutes over the left or right DLPFC.

**Results:**

We show that external modulation of both, the left and the right, DLPFC directly influences driving behavior. Excitation of the DLPFC (by applying anodal tDCS) leads to a more careful driving style in virtual scenarios without the participants noticing changes in their behavior.

**Conclusion:**

This study is one of the first to prove that external stimulation of a specific brain area can influence a multi-part behavior in a very complex and everyday-life situation, therefore breaking new ground for therapy at a neural level.

## Background

Standardized so-called "gambling tasks" in which participants can win or loose money by drawing cards from different decks have become an established tool for the investigation of "risk behavior" in psychological and neurophysiological research [Iowa Gambling Task:. [1], Cambridge Gambling Task: [2,3]]. Typically, riskier behavior in these tasks leads to higher gains but also to higher losses. The standardization of such gambling tasks is crucial when considering their clinical application; e.g. in the diagnosis of patients having problems with impulsiveness or planning and decision-making.

At a neural level, risk-taking behavior, decision-making and impulsiveness share similar neural networks in the dorsolateral prefrontal cortex (DLPFC) [1]. Patients with lesions in the DLPFC (especially in the right hemisphere) show riskier behavior than a healthy control group [4]. By contrast, lesions in the ventro-medial prefrontal cortex lead to "myopia" for the future, that is, insensitivity for future consequences of present behavior [1]. Interestingly, recent studies have shown that external stimulation of the DLPFC with Transcranial Magnetic Stimulation (TMS) [5] and with Transcranial Direct Current Stimulation (tDCS) [6] can influence risk-taking behavior.

The DLPFC is a brain region that matures through to late adolescence [7], and even during the second decade of life [8]. The late myelination of the DLPFC may serve as one possible explanation why adolescent behavior is often characterized by motivational difficulties, addiction and impulsivity [9]. The fact that driving accidents are the main cause of death for adolescents and young adults is a problem of paramount importance, also from a political perspective [10]. Different studies reported that risky driving behavior is more prominent among young drivers [11]. The frequency of substance abuse and the degree of aggressiveness are (besides gender and social factors) the main predictors for risky driving [12]. Furthermore, children diagnosed with ADHD have been shown to have an elevated risk for driving-related problems in adulthood [13]. In view of the preceding, we can assume that the DLPFC is importantly involved in modulating risky driving behavior. Results from the standardized "Risk-" and "Gambling-Tasks" are consistent with the findings about the neurodevelopment of the DLPFC, but the generalization of findings to everyday life situations is hampered by the high specificity of these paradigms.

The aim of this study was to examine the role of the DLPFC in a situation more closely associated with risk taking in everyday life. We hypothesized that excitation of the DLPFC causes stronger executive control and less risky driving behavior. In order to test this hypothesis, we modulated neural excitability of the participants' DLPFC before measuring their performance in a driving simulator. As mentioned above, several studies have reported differential involvement of the right and left DLPFC in the control of risk behavior [14]. Therefore, we also tested for such laterality effects expecting stronger effects after modulation of the right PFC.

In contrast to an earlier study on the modulation of risk behavior by external brain stimulation [5], we used tDCS instead of TMS. tDCS has the advantage that the participants barely notice the stimulation. Furthermore, depending on the direction of the current flow, neural excitability can be either enhanced or decreased.

## Methods

### Subjects

Twenty-four male subjects participated in the study. Twenty-one of them were students. All participants were between 20 and 30 years old (mean age: 24.1; SD: 2.7). Male subjects were chosen because in pilot experiments men were found to have a lower probability of experiencing nausea in the driving simulator. All of the participants were right-handed, had no history of neurological or psychiatric diseases and were in the possession of a driver's license for at least 2 years. The experiment was approved by the local ethic committee (ethic committee of the canton of Zurich, specialized subcommittee for psychiatry, neurology and neurosurgery, Oetwil am See, Switzerland).

### Experimental design

Every subject was tested on two different days within a week. On the first day, after a theoretical instruction about the driving simulator and the tDCS procedure, all subjects gave their written informed consent for participation in the experiment and filled in a questionnaire about their driving behavior (frequency of driving and years in possession of driver's license), education and health. Before the actual experiment, participants had the opportunity to drive a sample course ("circuit") in the driving simulator in order to get used to the simulation.

For the actual experiment, a course called "Mountain Course" [15] was chosen (see below for details). Every participant drove this course once without any tDCS influence. After this "baseline-drive", tDCS was applied over the DLPFC for 15 min (see below for details). In half of the subjects stimulation was applied to the right hemisphere, while in the other half the left hemisphere was stimulated. After the stimulation, the tDCS equipment was removed and the subjects drove the same course under the after-effect of the stimulation without cables on their heads. This protocol (testing performance after the application of tDCS) was used to increase external validity of the driving situation and relies on the long-lasting after-effects of tDCS (on the motor cortex until 90 min) [16]. To our knowledge, there are no studies that investigated after-effects of tDCS on prefrontal cortical areas. Therefore, we assume similar temporal characteristics as reported for the motor cortex. During the stimulation with tDCS, the participants sat outside the driving simulator on a chair and filled in the handedness and health questionnaires [17]. To assess subjective involvement in the virtual environment, an adapted version of the MEC-SQ (spatial presence questionnaire) [18] was filled in by the participants after each driving session. The possible impact of the tDCS stimulation on the emotional state was assessed using the Self-Assessment-Manikin [19] before and after stimulation. On the second day, the same procedure was applied but the stimulation electrodes were switched resulting in a stimulation condition (anodal or cathodal stimulation) different from that applied during the first experimental day. The order of the stimulation conditions was counterbalanced. The questionnaires about health and handedness were not filled in on this second day. This difference in procedure on the second day, however, is unproblematic since the conditions were counterbalanced across subjects.

### Driving simulator

The driving simulator used in the experiment is an upgraded version of the F10PF-Model of the Dr.-Ing. Reiner Foerst GmbH [15]. The virtual environment was projected on three 61" videowalls (RP 61" ES LCD) [20]. The actual test-course, called "Mountain Course", consists of a car that can be driven on a road starting outside a small village, passing through the village with traffic lights, and then following a route through built up areas. The simulation automatically stopped after a covered distance of 3 km (lasting around 3.5 min depending on driving speed). The scene was identical for each subject. Traffic, traffic lights, dangerous events (children, animals) etc. were simulated randomly in order to enhance the reality of the scene. Every 20 ms, about 30 measures with which to capture driving behavior were registered (e.g. driving speed, distance to driver ahead, position in the course, position of break, accelerator, gear, revolutions per minute, lateral distance from road mid-line etc.)

### Transcranial direct current stimulation

The "DC-Stimulator" distributed by neuroConn^© ^[21] was used for transcranial direct current stimulation. The constant current was applied through two saline-soaked electrodes with a surface of 35 cm^2^. Based on earlier studies modulating DLPFC excitability [5], stimulation electrodes were placed at the F3 or F4 position (international EEG 10–20-system), respectively for left and right-hemispheric stimulation. For DLPFC excitation, the anode was positioned on F3 (or F4) and the cathode was mounted on the ipsilateral mastoid. For DLPFC inhibition, the two electrodes were switched (cathode over F3/F4, anode over ipsilateral mastoid). The subjects were randomly divided into two equally sized groups. One group was stimulated on the left, the other on the right hemisphere. tDCS was applied for 15 minutes with a constant current intensity of 1mA. As a precaution measure, the "DC-Stimulator" automatically turns off when electrical resistance is too high.

### Statistical analysis

Dependent variables reflecting driving performance under the influence of tDCS were compared with the performance during the baseline-drive (before tDCS application) using repeated-measures ANOVAs with 'time' (2 levels; pre- vs. post-simulation) and 'stimulation condition' (2 levels; anodal vs. cathodal) as within-subject factors and 'side of stimulation' as between-subject factor. For each dependent variable ("distance to driver ahead", "driving speed", "speed violations" and "revolutions per minute") an individual ANOVA model was set up. Post-hoc t-tests (based on the Bonferroni-Holm procedure) were calculated to further explore the effects of the ANOVA.

## Results

**Figure 1:**
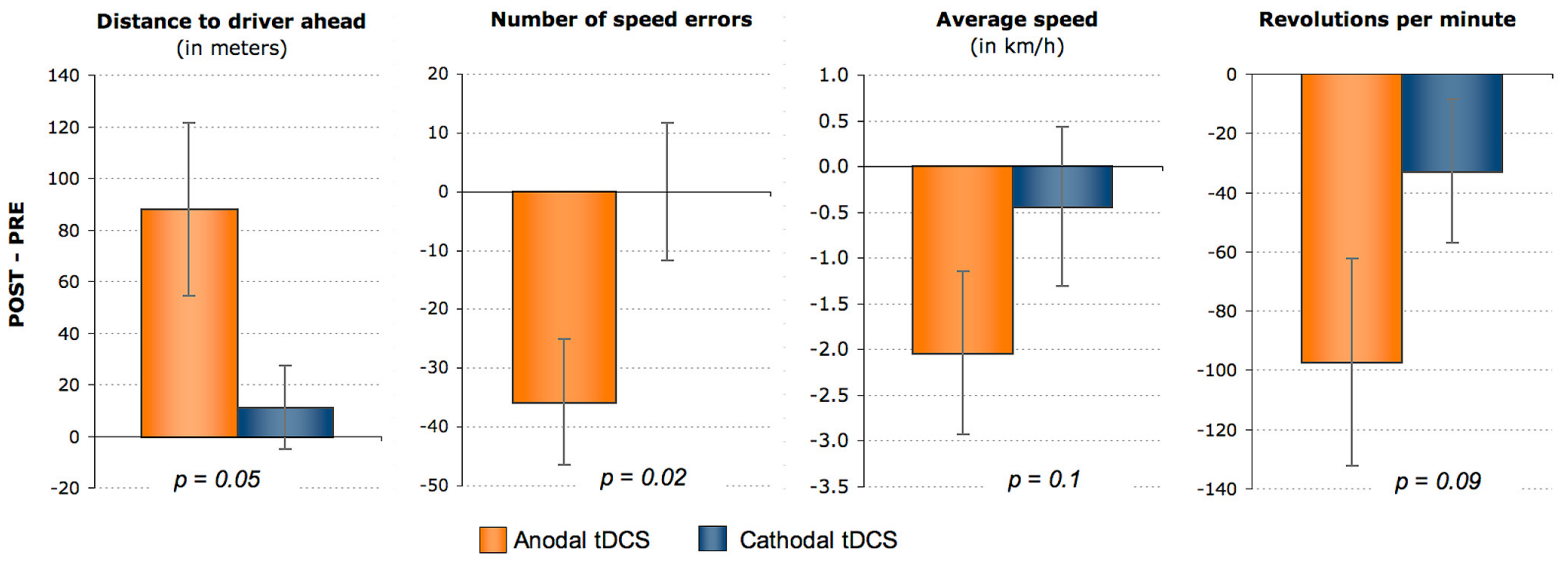
**Differences between anodal and cathodal tDCS**. Depicted are differences and standard errors (SE) between pre- and post-stimulation driving behavior (POST minus PRE) pooled across the two experimental groups (left DLPFC and right DLPFC stimulation). The p-values indicate the significances of the 'time × condition' interactions for each of the four behavioral variables.

Prior to the statistical analyses, behavioral data of three participants were excluded (right-hemispheric stimulation group: 1, left-hemispheric stimulation group: 2) because these subjects demonstrated extremely high or low values for the parameter "distance to driver ahead" before tDCS stimulation. The remaining data were subjected to repeated-measure ANOVAs with 'time' and 'stimulation condition' as within-subject factors and 'side of stimulation' as between-subject factor. As displayed in figure 1, the analyses revealed 'time × condition' interactions for "distance to driver ahead" *[F(1,19) = 4.25, p = 0.05] *and "number of speed violations in built-up areas" *[F(1,19) = 5.97, p = 0.02]*. The same trend was evident for "driving speed" *[F(1,32) = 2.83, p = 0.1] *and "revolutions per minute" *[F(1,32) = 3.21, p = 0.09]*. There was no main effect of 'side of stimulation' or an interaction of this between-subject factor with the variables of interest (time, condition) for any of the four variables.

Post-hoc paired t-tests revealed that anodal tDCS induced an increase of the "distance to driver ahead" *[right-hemisphere tDCS: T(10) = -1.77, p = .05; left-hemisphere tDCS: T(9) = -1.84, p = .05] *and declines in "number of speed violations" *[right-hemisphere tDCS: T(10) = 3.26, p = .005; left-hemisphere tDCS: T(9) = 1.54, p = .08]*, "driving speed" *[right-hemisphere tDCS: T(10) = 1.64, p = .07; left-hemisphere tDCS: T(9) = 1.56, p = .08]*, and "revolutions per minute" *[right-hemisphere tDCS: T(10) = -1.51, p = .08; left-hemisphere tDCS: T(9) = 3.10, p = .006]*. All four variables indicate a more cautious driving behavior when DLPFC activity is enhanced. For cathodal tDCS, on the other hand, only one trend was registered (decrease of "driving speed" in the left-hemisphere stimulation group; p = .08). Hence, learning effects induced merely by the repeated exposure to the task cannot explain the effects found.

**Figure 2:**
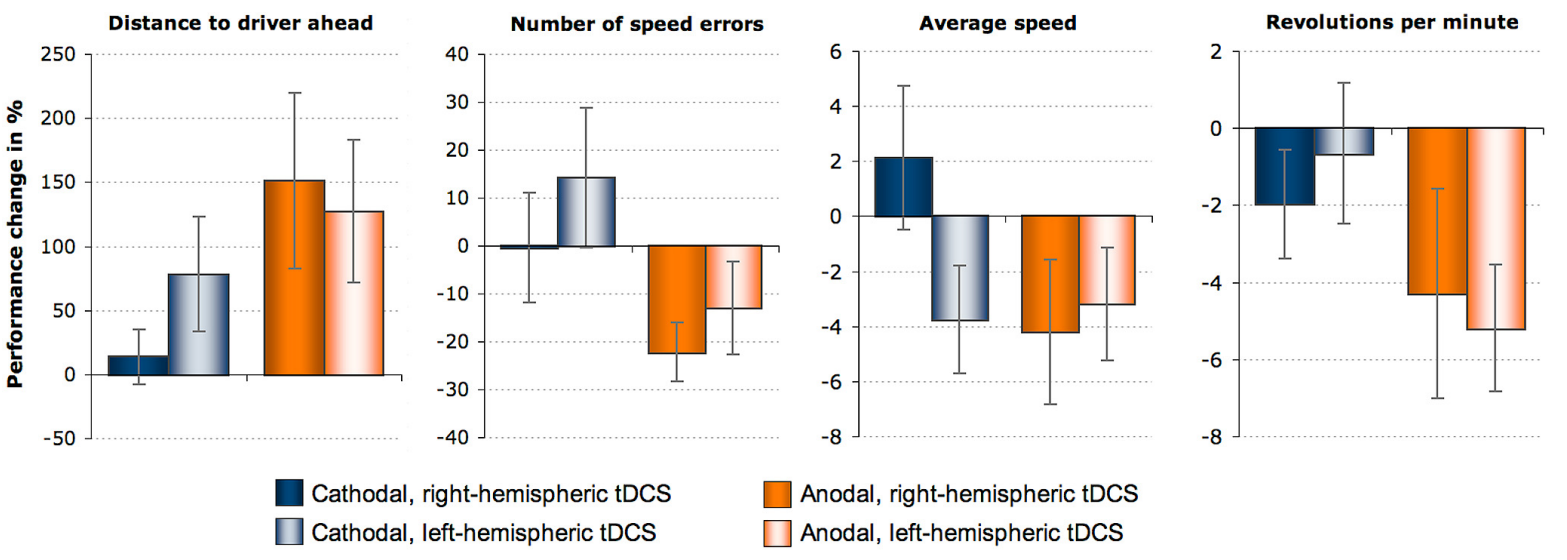
**Left- vs. right-hemispheric DLPFC stimulation**. Depicted are performance changes from pre- to post-stimulation measurements in percent ((POST*100)/PRE) and standard errors (SE) separately for two experimental groups (left DLPFC and right DLPFC stimulation).

To compare the two groups, post-stimulation performance was related to the individual pre-stimulation performance for each participant (a posttraining value of 100% means no change from pre- to posttraining). The corresponding values are depicted in figure 2. Since, in case of cathodal stimulation, the resulting values did not differ significantly from the reference value (100%), we refrain from comparing between-group differences. With respect to anodal tDCS, two-sample t-tests comparing the performance changes between the two groups resulted in p-values > 0.4 for all behavioral variables.

Subjects did not indicate different degrees of presence in the virtual scene in the different conditions, and there were no differences in emotion either, as reported with the Self-Assessment Manikin. None of the subjects reported nausea during driving simulation. The years in possession of a driver's license were not associated with different effects of tDCS.

## Discussion

The present study aimed to examine effects of tDCS, and hence, of the induced manipulation of DLPFC activity on driving behavior in a customary driving simulator. As a main result, we found that anodal tDCS evoked less risky driving behavior while cathodal tDCS did not significantly influence the driving style. With respect to anodal stimulation, behavioral differences were found in four variables ("driving speed", "distance to driver ahead", "number of speed violations", "revolutions per minute") measuring different aspects of driving behavior. While the "distance to driver ahead" was larger after anodal tDCS as compared to the baseline measurement, the "number of speed violations", the "driving speed" and the "revolutions per minute" were reduced. These strong behavioral changes are evident despite the fact that the participants were not aware of their change in behavior.

The crucial association between functions mediated by the prefrontal cortex and risk-taking driving behavior found in this study is in line with previous findings about the prefrontal cortex [1]. It is remarkable that a complex behavior such as driving a car can be directly influenced by an external modulation of the cortical excitability. Our main result is consistent with earlier research focusing on the external modulation of DLPFC activity and its effects on risk-taking behavior in situations less closely related to risk-taking in everyday life [5,22,23]. Knoch et al., for example, showed that low-frequency rTMS applied over the DLPFC evoked more risky behavioral choices in a standard gambling paradigm [5]. The authors did not, however, study the effect of DLPFC excitation (as evoked by high-frequency rTMS) in their study, which would have induced similar effects in the stimulated tissue as anodal tDCS. Furthermore, Fecteau et al. reported a reduction of risk-taking behavior in different task paradigms following tDCS applied over the DLPFC [6,24]. In their studies, the two electrodes were mounted to overly the left and right DLPFC areas – an electrode configuration that allowed the simultaneous stimulation of both hemispheres. Depending on the task, behavioral effects were evident only after right anodal/left cathodal stimulation [24] or after both, right anodal/left cathodal and left anodal/right cathodal DLPFC stimulation [6]. Using EEG combined with the estimation of intracerebral sources of brain activation, a recent study of our group uncovered less activation in the right-sided DLPFC during speeded and impulsive driving [25]. This finding is in close correspondence with the study by Clark et al. [4] reporting the same lateralization effects in patients with brain lesions in the context of risk behavior and with the later study of Fecteau et al. [24]. The present study, however, did not replicate this lateralization effect and, thus, rather supports the earlier study of Fecteau et al. [6]. Considering the data currently available, we have to conclude that the issue of functional DLPFC lateralization in the context of risk-taking behavior is not entirely understood – studies comparing simultaneous stimulation protocols as used by Fecteau et al. with stimulation protocols where the reference electrode is positioned on a functionally ineffective position would contribute to clarify this issue.

The propensity of risk-taking behavior has been assumed being linked to the openness to drug experiences and the general vulnerability for pathological addictive behavior. Several previous studies demonstrated that noninvasive stimulation of the frontal cortex lessens the craving for typical drugs such as nicotine [26,27] or cocaine [28], hence suppressing the need to initiate reward-related behavior. In a broader sense, this effect corresponds to a more deliberate style of behavior and is consistent with the main result of the present study.

One may argue that the lowering of the driving speed can be explained by a stimulation-induced decline of attention. However, since anodal tDCS stimulation is known to enhance neural excitability in cortical regions underlying the stimulation electrode, it seems unlikely to us that anodal stimulation reduces attentional capacities (if anodal tDCS should modulate attention we would anticipate increased and not decreased attentional functionality). It can be further speculated about general effects of boredom or tiredness after the stimulation break; however, this effect should be the same after anodal and cathodal stimulation and can therefore not explain the observed difference between stimulation conditions. In addition, the increased carefulness of driving that we observed following anodal tDCS was not only characterized by a generally slower driving speed but also by a reduced number of speed violations, by reduced revolutions per minute and by an enlarged distance to the car driving ahead. In our opinion this combination of effects points to a more careful driving style rather than to enhanced tiredness.

Cathodal stimulation did not lead to a significant alteration of driving behavior. The reasons for this lacking effect are difficult to explain, thus we can only offer more or less speculative explanations. Given that functional lateralization of the DLPFC is an unsolved issue, it may well be that the unilaterally evoked hyperpolarization is simply not strong enough to induce a clear behavioral effect. The hemisphere not stimulated may be equipped to solely prevent the individual from showing more risky behavior – a mechanism that would be reasonable from an evolutionary perspective. Alternatively, it may be argued that the mere notice of receiving electrical stimulation (perceived as a slight itching at the beginning of the stimulation) leads to more careful driving that is independent of the stimulation condition. This effect may have counteracted a potential enhancement of a risky driving style induced by cathodal stimulation and on the other hand it may have facilitated the behavioral effect of anodal tDCS.

There is one methodological limitation of the tDCS technique that should be addressed. Given the electrode size of 35 cm^2^, it is obvious that the spatial resolution is low. Furthermore, when using this technique, one has to deal with remote effects. Since the brain is a heavily wired system, current spread from the stimulated region to neighboring and interconnected regions is most likely. Remote effects have been proven in studies combining transcranial brain stimulation (TMS, tDCS) with PET or fMRI [29,30]. With respect to the present study, it may be that the stimulation of the DLPFC triggered a co-activation of other regions in the frontal lobe such as the ventromedial or orbitofrontal cortex, which may have influenced task performance after stimulation. Since we do not know the real extent of the tDCS effect, we cannot disentangle precisely the neurophysiological underpinnings and the associated psychological processes. Although several methodological problems of tDCS are unsolved so far, there are several studies available supporting the precision and usability of tDCS [31,32]. Uy and Ridding, for example, showed a specific increase of cortical excitability for the First Dorsal Interosseous (FDI) muscle after anodal tDCS while the Abductor Digiti Minimi (ADM) and the Flexor Carpi Ulnaris (FCU) were not affected. However, it was not in the scope of this study to clarify the exact neuroanatomical source of the effect. Further studies combining tDCS with neuroimaging methods are needed to address this issue properly.

It is important to highlight the significance of the present findings for the explanation of aggressive and risky driving behavior, especially in adolescents. The stimulation of the DLPFC influenced driving behavior. Exactly this site of the brain matures late in adolescents and might be the cause of deviant driving styles. Regardless of the high behavioral complexity in this paradigm, we found striking results with high external validity and direct transferability in everyday life. Moreover, the feasibility of external manipulation of the brain, even on complex behaviors, opens different possibilities for neural rehabilitation.

## Competing interests

The authors declare that they have no competing interests.

## Authors' contributions

GB participated in the design of the study, performed parts of the statistical analysis and drafted the manuscript. KG participated in the design, carried out the experiments and performed the statistical analysis. SK participated in the design. LJ participated in the design, the statistical analysis and helped drafting the manuscript. All authors read and approved the final manuscript.
